# Acute Respiratory Distress Syndrome: Mechanical Ventilation vs. High‐Frequency Percussive Ventilation

**DOI:** 10.1155/carj/5796675

**Published:** 2026-07-28

**Authors:** Daniel Sagan, Carol Parise, Imran Mohammed, Amandeep Mann, Mehreen Mohammed, Abdullah Alismail

**Affiliations:** ^1^ Department of Cardiopulmonary Sciences, School of Allied Health Professions, Loma Linda University Health, Loma Linda, California, USA, lluh.org; ^2^ Cardiopulmonary Department, Sutter Health, Sutter Roseville Medical Center, Roseville, California, USA, sutterhealth.org; ^3^ Sutter Center for Health Systems Research, Sutter Health, Sacramento, California, USA, sutterhealth.org; ^4^ Pulmonary Medicine Associates, Sacramento, California, USA; ^5^ Department of Medicine, School of Medicine, Loma Linda University Health, Loma Linda, California, USA, lluh.org; ^6^ Research and Innovation Institute, Ministry of Defense Health Services, Riyadh, Saudi Arabia

**Keywords:** ARDS, high-frequency percussive ventilation, mechanical ventilation

## Abstract

**Background:**

Studies that investigate the use of high‐frequency percussive ventilation (HFPV) are scarce and outdated. HFPV is being used in some facilities primarily as a salvage modality on patients with acute respiratory distress syndrome (ARDS) when a conventional mechanical ventilator has failed to improve outcomes. There still are arguments whether HFPV is an effective ventilation strategy for adult patients with ARDS.

**Objectives:**

The purpose of this study was to analyze and compare pulmonary gas exchange, as measured by arterial blood gas (ABG) values, between patients with ARDS who were transitioned from conventional mechanical ventilation to HFPV and those who remained on conventional mechanical ventilation.

**Methods:**

This was a retrospective study between 2016 and 2022. Data was extracted and analyzed from electronic medical records (EMRs) of ARDS patients. ABG results were analyzed from 12 to 2 h before, and 30 min, 2, 12, and 24 h after the initiation of conventional and HFPV. Continuous mandatory ventilation (CMV) settings were compared over 12 and 2 h before the initiation of HFPV and 2, 12, and 24 h after.

**Measurements and Main Results:**

A total of *N* = 351 cases were evaluated for this study (*n*1 = 194 in the CMV group and *n*2 = 157 in the HFPV group). Patients in the HFPV group had a lower mean baseline pH (7.25 vs. 7.34, *p* = <0.001) and higher PaCO_2_ (56.7 vs. 40.3 mmHg, *p* = <0.001) when compared to the CMV group. pH has increased and PaCO_2_ decreased quickly once HFPV was initiated. The mean baseline PaO_2_ was comparative (61.7 vs. 67.4 mmHg, *p* = 0.21) but the HFPV group had a more rapid and higher improvement compared to the CMV group (121.5 vs. 86.6, *p* = <0.001). Similarly, there was a significant improvement in the PF ratio after 12 h on HFPV (160.7 vs. 139.7, *p* = 0.03).

**Conclusions:**

Results show that HFPV can effectively be used for adult ARDS as a salvage modality when a patient’s ABG has become refractory to conventional mechanical ventilation.

## 1. Introduction

The global severe acute respiratory syndrome coronavirus 2 (SARS‐CoV‐2) pandemic has reaffirmed the difficulty of treating patients who suffer from acute respiratory distress syndrome (ARDS) [1]. Mortality remains high and survivors frequently suffer chronic complications [2, 3]. Intubation and mechanical ventilation are the most common intensive care unit (ICU) interventions for severe ARDS when noninvasive measures fail [4, 5].

Standard strategies for protecting the lungs of ARDS patients involve using ventilation with restricted tidal volume (6 mL/kg) and maintaining limited plateau pressures (< 30 cmH_2_0) as per the standard of care [6, 7]. In addition, another frequently used approach is alveolar recruitment maneuvers with increases in positive end–expiratory pressures (PEEPs) that have shown reduced hospital and 28‐day mortality [8, 9]. Evidence suggests that prone positioning patients with ARDS reduces ventilator‐induced lung injury by promoting uniform lung stress and strain distribution and improves oxygenation [10, 11]. Unfortunately, patients frequently become refractory to conventional ventilatory techniques, and gas exchange remains unaltered or worsens and thus requires unconventional salvage strategies to ventilate the injured lungs [12, 13]. Other salvage techniques include the use of inhaled nitric oxide, extracorporeal membrane oxygenation (ECMO), airway pressure release ventilation (APRV), and high‐frequency ventilation (HFV).

One of the strategies in treating these patients is using high‐frequency percussive ventilation (HFPV). It is an advanced ventilation strategy that can be considered as a salvage option in this patient population, as it already has been proven to improve gas exchange with success in trauma [14, 15], smoke inhalation [16, 17], weaning obese patients [18], pediatric respiratory failure [19, 20], and weaning off ECMO [21]. However, HFPV is one HFV technique that has not been widely studied in the adult ARDS patient population. It is crucial not to confuse high‐frequency oscillatory ventilation (HFOV) with HFPV, as two clinical trials in 2013 demonstrated unfavorable outcomes of HFOV in adult patients with ARDS [22, 23].

Currently, the only modality available for HFPV administration is the Volumetric Diffusive Respirator (VDR‐4, Percussionnaire, Bird Technologies based out of Sandpoint, ID, USA). There are no randomized controlled studies to date on HFPV, but there have been smaller retrospective studies dating back to late 1980s when HFPV was first introduced. There was no significant difference in mortality rates between the HFPV and control groups in the initial clinical trials conducted in 1989 and 1990; however, there was an improvement in oxygenation and ventilation at a lower mean and peak airway pressures in the HFPV group [24]. Still, it is unknown whether there are better patient outcomes between the HFPV groups and the conventional mechanical ventilator groups, particularly when it is used as a salvage modality.

The purpose of this study was to analyze and compare gas exchange in the lungs via the arterial blood gas (ABG) values between ARDS patients placed on a mechanical ventilator and subsequently moved to HFPV with patients who remain on conventional mechanical ventilator.

## 2. Methods

This study was approved by Sutter Health Institutional Review Board. The electronic medical record (EMR) was used to query adult patients admitted between January 1, 2016, and May 31, 2022, who met the Berlin definition of ARDS, had a moderate‐to‐severe PaO_2_/FiO_2_ ratio (PF ratio < 200), were in the adult medical ICU, and were mechanically ventilated during their entire ICU (CMV group) stay or were transferred from a mechanical ventilator to a VDR (HFPV group). Our study facilities have no protocol for the initiation of HFPV, hence HFPV was initiated solely based on physician preference. Once HFPV was initiated, facility protocols were then used to make setting adjustments to the ventilator based on the patient’s ABG. These facility protocols are based on the recommendations by the manufacturer. The study excluded patients admitted for trauma and postoperative care. ABG readings were obtained 12 and 2 h before HFPV and conventional mechanical ventilator placement. In addition, the following ABG values were extracted: 30 min and 2, 12, and 24 h following the initiation of HFPV and conventional mechanical ventilator.

### 2.1. Statistical Analysis

The data were screened and cleaned using frequency distributions, histograms, and descriptive statistics to identify data outliers and document missing values. Differences between the two groups (HFPV vs. CMV) on the demographic and clinical characteristics at ICU admission were analyzed using analysis of variance for continuous variables and the *X* ^2^ test of independence for categorical variables.

Statistical analysis was conducted using repeated measures’ mixed modeling. Mixed models’ repeated measures account for the correlations among the responses of the same subject over repeated time points and allow for the use of all observations available for a time point. For each ABG, the initial model building process included the fixed effects of ventilator type (mechanical ventilation, HFPV), time (6 time periods), age, weight at admission to the ICU, and the time *X* ventilator type interaction. Variables that did not contribute to model fit were subsequently removed. Mixed models were also used assess the repeated measurements of CMV over the 6 time periods. An unstructured covariance structure was used. Post hoc tests were adjusted using the least significant difference procedure. All analyses were conducted using IBM SPSS 29.0.

## 3. Results

**FIGURE 1:**
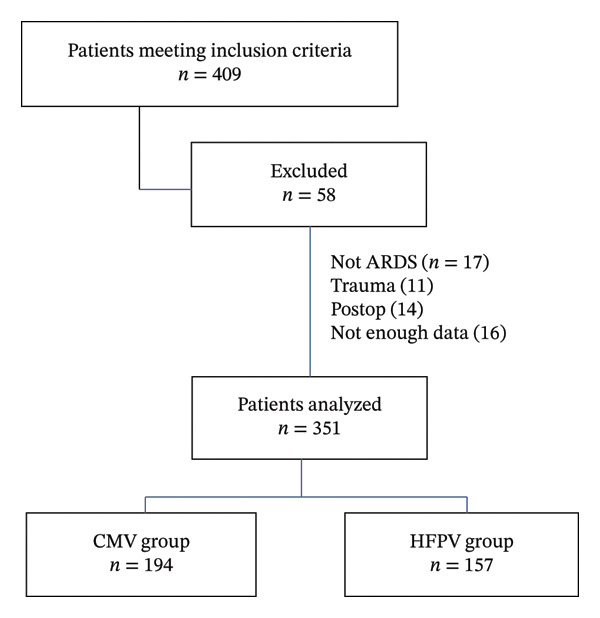
Flowchart of patient selection criteria.

In total, 409 patients met the criteria for inclusion. Fifty‐eight patients were excluded (40 in the CMV group and 18 in the HFPV group), leaving a total of 194 patients in the CMV group and 157 in the HFPV group (Figure 1).

Table 1 presents the demographic and clinicopathological characteristics of the study population. The majority were males, of which 92 transitioned to HFPV. Patients ventilated via HFPV were younger but weighed more and had a slightly higher BMI. There were 136 patients who had ARDS due to COVID‐19. Statistically significantly fewer patients with COVID‐19 were placed on HFPV versus those who remained on a mechanical ventilator (*n* = 43, 27.4%, vs. *n* = 93, 47.9%, *p* = <0.001). There were no statistically significant differences (*p* = 0.13) in mortality between the HFPV (100 died out of 157, 63.7%) and CMV groups (120 died of 194, 61.9%). Notably, mortality was 66.9% among patients in moderate‐to‐severe ARDS with COVID‐19.

**TABLE 1:** Demographic characteristics at ICU admission.

	Mechanical ventilator *n* = 194	HFPV *n* = 157	*p*
	Mean ± SD	Mean ± SD	
Age	56.42 ± 15.81	52.83 ± 16.17	0.04
APACHE score	82.66 ± 36.04	77.32 ± 39.07	0.07
Weight	197.00 ± 61.63	215.38 ± 74.51	0.02
Body Mass Index	31.29 ± 9.97	33.99 ± 10.72	0.04

	***n* (%)**	***n* (%)**	**p**

Sex			0.46
Male	106 (54.6)	65 (41.4)	
Female	88 (45.4)	92 (58.6)	
Asthma	7 (3.6)	18 (11.5)	0.004
COPD	15 (7.7)	16 (10.2)	0.420
CAD	11 (5.7)	14 (8.9)	0.240
Hypertension	61 (31.4)	56 (35.7)	0.404
Diabetes	51 (26.3)	35 (22.3)	0.387
ESRD	10 (5.2)	10 (6.4)	0.625
COVID‐19	93 (47.9)	43 (27.4)	< 0.001
Cancer	19 (9.8)	14 (8.9)	0.780
Muscle relaxants	176 (90.7)	90 (94.7)	0.236
Prone positioning	58 (29.8)	67 (42.7)	
Mortality	120 (61.8)	100 (63.7)	0.125

Abbreviations: CAD, coronary artery disease; COPD, chronic obstructive pulmonary disease; ESRD, end‐stage renal disease.

**FIGURE 2:**
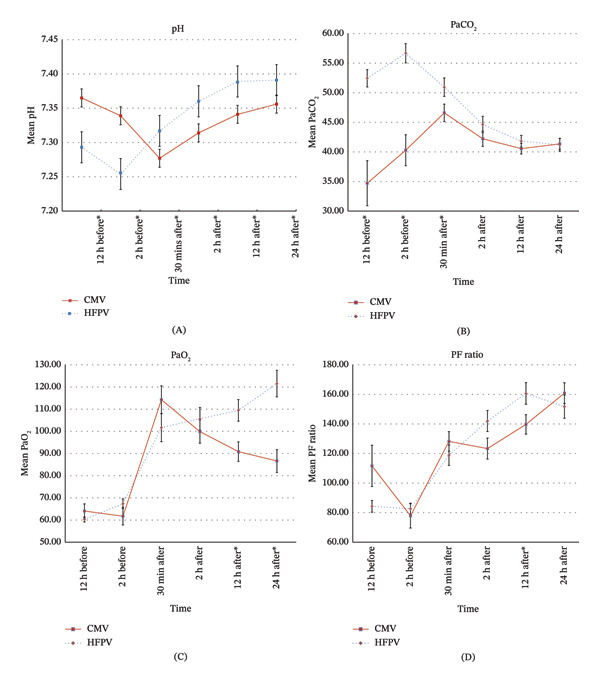
Patients with moderate to severe ARDS transferred to HFPV (blue line) and remaining on a conventional mechanical ventilator (red line). Panel (A) pH was statistically significantly (*p* < 0.05) higher for patients in the CMV group 12 and 2 h prior to mechanical ventilator and higher (less acidotic) for the HFPV group starting 2 h after and beyond. Panel (B) The HFPV group had statistically significantly (*p* = <0.001) higher PaCO_2_ than patients in the CMV group 12 and 2 h before and 30 min after placement. There was no statistically significant difference between the two groups after 24 h (*p* = 0.91). Panel (C) PaO_2_ was statistically significantly (*p* < 0.01) higher (better oxygenation) in the HFPV at 12 and 24 h after placement than patients in the CMV group. Panel (D) There were no statistically significant differences (*p* > 0.05) in the PF ratio between HFPV and CMV.

Figure 2 shows the means and standard deviations of the ABG values at each time of measurement. The post hoc tests resulting from the analyses showed statistically significant (*p* < 0.05) differences in mean pH between the CMV and HFPV groups at all time points. The pH was higher for patients in the CMV group 12 and 2 h prior to mechanical ventilator placement and higher (less acidotic) for the HFPV group starting 2 h after and beyond (Panel A). Patients in the HFPV group had statistically significantly (*p* = <0.001) higher PaCO_2_ than patients in the CMV group 12 h and 2 h before and 30 min after placement. Reduced PaCO_2_ was observed in the HFPV group, with no statistical difference between the two groups after 24 h (*p* = 0.91) (Panel B). The only differences in PaO_2_ were at 12 and 24 h after placement, where patients in the HFPV group had statistically significantly (*p* < 0.01) higher PaO_2_ values (better oxygenation) than patients who remained on a mechanical ventilator (Panel C). Patients in the HFPV group had higher PF ratios at 2 and 12 h after placement, but these differences were not statistically significant (*p* > 0.05) (Panel D).

Table 2 shows the mean ventilator parameters before and after the initiation of HFPV. There were no differences across any of the time points for PIP. PIP 24 h after the initiation of HFPV was statistically significantly higher than 2 and 12 h prior to initiation. Twenty‐four h after the initiation of HFPV, PEEP at was statistically significantly (*p* < 0.05) lower than 2 h after and higher than 2 h and 12 h prior to initiation.

**TABLE 2:** Mean ventilator parameters before and after the initiation of HFPV.

	12 h	2 h	2 h	12 h	24 h
	Mean ± SD	Mean ± SD	Mean ± SD	Mean ± SD	Mean ± SD
	Before initiation of HFPV		After initiation of HFPV		
PIP	33.61 ± 6.53	34.03 ± 6.07	32.70 ± 5.61	33.91 ± 7.06	33.50 ± 7.30
MAP	19.06 ± 5.19	20.00 ± 4.57	23.58 ± 4.22	24.24 ± 4.75	24.01 ± 5.42
PEEP	11.46 ± 3.93	12.09 ± 4.24	15.18 ± 3.92	14.63 ± 3.78	14.57 ± 4.24
FiO_2_	80.51 ± 21.50	88.19 ± 18.71	82.51 ± 19.48	72.72 ± 20.68	67.20 ± 21.08
OI	25.60	26.18	18.41	16.10	13.28

	Before initiation of CMV		After initiation of CMV		
PIP	—	—	28.59 ± 7.19	27.61 ± 6.82	26.79 ± 6.27
MAP	—	—	16.53 ± 4.72	15.94 ± 4.70	15.63 ± 4.74
PEEP	—	—	10.73 ± 3.79	10.69 ± 3.69	10.58 ± 3.49
FiO_2_	78.16 ± 22.88	86.73 ± 19.91	82.06 ± 19.62	72.42 ± 20.93	66.74 ± 20.68

*Note:* PIP: peak inspiratory pressure (measured in cmH_2_0). MAP: mean airway pressure (measured in cmH_2_0). PEEP: positive end–expiratory pressure (measured in cmH_2_0).

Abbreviations: FiO_2_, fraction of inspired oxygen; OI, oxygen index.

**FIGURE 3:**
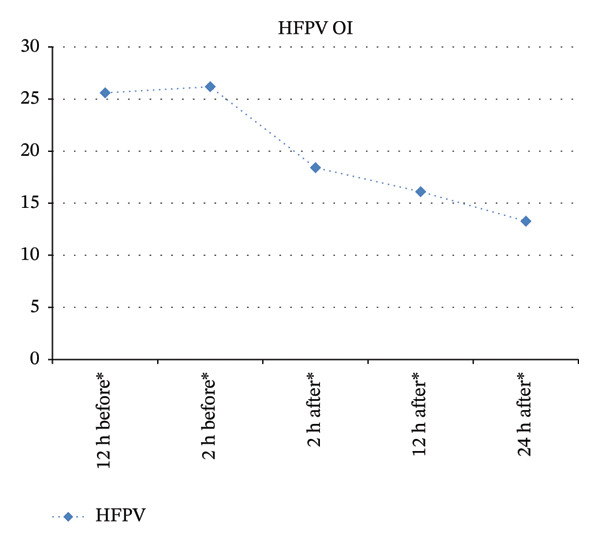
OI before and after HFPV. Values at 24 h of HFPV were significantly lower than those at all other time points (*p* < 0.01).

Figure 3 shows that the oxygen index (OI) was reduced after the initiation of HFPV, decreasing from 26 to 13 within 24 h. This difference was statistically significant (*p* < 0.01).

## 4. Discussion

The results of this study demonstrate that gas exchange is more efficient during HFPV. Acid–base balance improved significantly and stabilized within normal ranges following the initiation of HFPV. Notably, prior to initiation, the HFPV group exhibited a more acidotic pH compared with the CMV group; however, after HFPV was initiated, pH normalized within 30 min, whereas a comparable improvement required approximately 2 h in the CMV group.

In patients with moderate‐to‐severe ARDS, it is quite common for clinicians to allow “permissive hypercapnia” with a pH of 7.20 as nonconcerning, as long as the key strategies emphasize low tidal volumes and limiting peak and plateau pressures [25]. Studies of permissive hypercapnia are usually confounded by the inability to separate between the effects of permissive hypercapnia and low tidal volume and have conflicting evidence on mortality. The HFPV group in the present investigation had a mean pH of 7.24 before the initiation of HFPV. Although some clinicians may not find this concerning, we wanted to highlight the increase of the pH after the initiation of HFV.

We also observed enhanced oxygenation with a decreasing OI, improved P/F ratio, and reduced FiO_2_ once HFPV was initiated. Although PaO_2_ and the P/F ratio improved significantly at 2 and 12 h following the initiation of HFPV, these improvements were no longer statistically significant at 24 h.

It is important to note that HFPV was used as a salvage modality in patients who were refractory to conventional mechanical ventilation and comparing the two groups is problematic due to differences in how ventilation modes are measured for several reasons. Patients in the CMV group were not invasively ventilated prior to initiation of CMV. We calculated the OI to account for the increase in mean airway pressures following HFPV initiation and despite this increase, OI continued to improve after HFPV was started. PIP displayed on the HFPV may be higher than what is shown on the digital multimeter on the machine and is not analogous to a peak inspiratory pressure in conventional ventilation. Dutta et al. observed that peak inspiratory pressures were higher in HFPV compared to pressure‐controlled ventilation, but time spent at the reported monitron PIP (0.12 s) is not equivalent to time spent at a conventional ventilator peak inspiratory pressure (I‐time 0.8–1.2 s) [26]. Despite these discrepancies, the primary objective of this study was to assess the gas exchange between the two different ventilators, which was successfully achieved.

HFPV has expiratory countercurrent flow generated from a high‐flow shock wave of inspiratory gas, clearing mucus plugs that obstruct the bronchi and have a constant outflow of CO_2_ gas [27]. Burn centers have adopted HFPV for mobilizing secretions caused by sloughing, subsequently improving gas exchange and perfusion [28]. Additionally, some neonatal and pediatric ICUs have adopted HFPV for children suffering from acute respiratory failure. The largest cohort of HFPV to date had a sample size of 193 children and demonstrated improved gas exchange after only 2 h on HFPV [20]. To our knowledge, the present investigation is the first one that assessed changes in gas exchange in over 300 adult patients with ARDS who were either only mechanically ventilated or mechanically ventilated and subsequently placed on HFPV.

The mechanism of gas exchange in HFV is not well understood but theories including pendelluft, Taylor dispersion, cardiogenic mixing, and coaxial, laminar, countercurrent flow have been proposed [29–32]. These theories introduce vastly different aerodynamics than the conventional mechanical ventilator, which delivers turbulent, bulk flow ventilation [29]. An increase in the PF ratio on HFPV patients is consistent with current literature. HFPV used on 16 patients post cardiac surgery who developed ARDS resulted in a statistically significant median P/F ratio increase of 62–169, which avoided the need for ECMO [33]. Multiple other studies also concluded that transitioning patients to HFPV leads to a significant, rapid, and sustained improvement in the P/F ratio [34–36]. Similar studies done in HFPV also saw an improvement in the PF ratio in the high‐frequency group compared to conventional mechanical ventilation; however, clinical outcomes showed no differences between the groups [23]. One study on HFV showed a higher mortality in the HFOV group, even though the PF ratio improved [22]. One of the limitations of a VDR is the inability to measure the plateau or even know the tidal volume delivered to the patient since it is an open circuit, flow ventilation that is believed to adapt to the compliance of the injured lung [36]. Allan and colleagues utilized a pneumotachograph to measure the tidal volume at different frequencies and found it to be over 1 L, which may be harmful for patients in ARDS [37]. The present investigation found that mean PIP remained unaltered at 33 cmH2O at all time points, which suggests that the PIP is similar whether patients are placed on HFPV or remain on a mechanical ventilator (Table 2). Even with similar pressures, the HFPV was able to normalize ABG values. This aligns with the current literature, with some case studies even reporting that oxygenation and ventilation have improved at lower peak inspiratory pressures [38]. Tidal volumes and important lung mechanics measurements, such as the compliance, were difficult to obtain retrospectively due to the varied results charted in the EHR. However, we have found it interesting that with the similar ventilator parameters between the two groups, the ABG results normalized in the HFPV group, which were refractory to the conventional mechanical ventilator.

This study has several limitations. ICUs do not use HFPV frequently because they are typically only available in large healthcare facilities and not all respiratory therapists or physicians are skilled or comfortable with the VDR. Because it is a complex ventilator to manage, many facilities modify ventilator protocols to reverse the refractory state of the patient or prevent the further decline of an unstable patient. It is possible that other facilities may modify their settings differently, which would limit the generalizability of these findings. In addition, this study utilized data from the EMR, a valuable resource for retrospective research. However, we acknowledge considerable variability regarding the placement of patients on HFPV based on decisions in the best interest of the patients. This introduces potential unknown biases that could not be controlled but is reflective of the real‐world experience of HFPV use in the ICU setting. Furthermore, there were other modalities in each group that might have altered the gas exchange in our study. Some patients were placed in a prone position, some on nitric oxide, and some were placed on ECMO. These modalities may have altered the gas exchange in particular patients but doubtful that it affected the overall results because of the number of cases included in the study. Several pulmonary and cardiac medications used in ARDS may independently affect gas exchange and oxygenation, including neuromuscular blocking agents, corticosteroids, bronchodilators, vasopressors, inotropes, and diuretics. Inhaled pulmonary vasodilators such as prostacyclin were not utilized at the study facilities, and nitric oxide is not common in the HFPV group. Variability in the use and timing of these therapies may have influenced oxygenation outcomes independent of ventilator modality.

In conclusion, HFPV is an effective salvage modality for adults with ARDS who develop ABG abnormalities refractory to conventional mechanical ventilation. Large, controlled trials are needed to validate these findings before adopting changes to the standard of care regarding the use of HFPV as a primary ventilation modality for adult ARDS.

## Author Contributions

Daniel Sagan came up with the research question, created the study design, created a literature review, and was a major contributor in the writing of the manuscript. Carol Parise contributed to the study design, analyzed and interpreted data, and was a major contributor to the manuscript. Imran Mohammed assisted with the research question, contributed to the study design, and interpreted the data. Amandeep Mann extracted the data and assisted with editing the manuscript. Mehreen Mohammed assisted with creating tables and editing of the manuscript. Abdullah Alismail supported the research question, assisted with the literature review, was a major contributor to the study design, interpreted analyzed data, and contributed to writing the manuscript.

## Funding

This was funded by grant 947110‐1107088 by the Sutter Medical Center Sacramento Foundation.

## Disclosure

All authors have read and approved the final manuscript.

## Ethics Statement

This study was approved by Sutter Health Institutional Review Board (IRB) as a retrospective study. Since this was a retrospective study, the informed consent was waived by the approving ethics committee. All methods were performed in accordance with relevant guidelines and regulations.

## Conflicts of Interest

The authors declare no conflicts of interest.

## Data Availability

All related data are included within the manuscript. To request access to the datasets, with a reasonable request, please email the corresponding author: aalismail@llu.edu.
